# Social determinants of health during and after coronavirus: a qualitative study

**DOI:** 10.1186/s12889-024-17785-7

**Published:** 2024-01-24

**Authors:** Farideh Izadisabet, Aliakbar Aminbeidokhti, Sakineh Jafari

**Affiliations:** 1 Doctoral student of educational management of Semnan University, Department of Midwifery, Faculty of Nursing and Midwifery, Semnan University of Medical Sciences, Semnan, Iran; 2 Department of Education Management, Faculty of Psychology and Educational Sciences, Semnan University, Semnan, Iran; 3 Department of Education Management, Faculty of Psychology and Educational Sciences, Semnan University, Central Administration of Semnan University, Campus 1, 35131-19111 Semnan, Iran

**Keywords:** Health, Social Factors Influencing Health, Coronavirus, Post-COVID

## Abstract

**Background:**

Health has multiple dimensions influenced not only by individual factors but also by broader social, economic, cultural, and political structures. The widespread COVID-19 pandemic has multidimensional effects on people’s lives, which can have effects on individuals’ lifestyles after the COVID-19. This study aimed to speculate the social determinants of health during and after the COVID-19, which can lead to more effective planning for promoting community health.

**Methods:**

The present study interviewed 21 experts in social and medical fields during four months. The sampling method was snowball. The interviews were semi-structured and administered in-person or electronic. All interviews were transcribed and analyzed according to the Brown and Clarke’s six-stage framework to extract themes.

**Results:**

the participants were 13 males, eight experts in social field, all had PhD, 17 were academic members, and 10 were members of the Social Determinants of Health Research Center. The qualitative content analysis induced seven different social themes that affect the health which included: justice (3 Subcategories), integration (4 Subcategories), acceptance (4 Subcategories), participation (2 Subcategories), adaptation (3 Subcategories), flourishing (4 Subcategories), and cohesion (3 Subcategories).

**Conclusions:**

According to the present study, a grand plan to cover all positive and negative social effects of COVID-19 should have at least seven different dimensions. However, the present models of effective social determinants in health do not have such comprehensiveness. Future studies may provide a proper model to be used in clinical and research fields.

## Background

Society is a group of people who live together and have various cultural, political, and economic interactions and exchanges, therefore the society has structure. Contemporary complex industrial societies and past simple societies have a common aspect: they are not simply the result of people coming together. But, each possesses its specific social structure, distinguishing them from other imagined social forms. The philosophy of social formation is based on the realization that only within a society, people can fulfill and satisfy specific fundamental needs or, at the very least, access them more easily [1]. Human societies are various interwoven factors including culture, social, economic, and politic. While these factors are distinct, they interact with each other, establishing tangible connections. The interactions of these factors can render the conditions of society critical and disordered or desirable and ideal. As creators of the constituents of human societies, humans assume various social roles, and the proper fulfillment of these roles enhances interactions within the community. Humans can effectively perform their social functions and continue their activities while they feel themselves healthy and their living environment is not disturbing their comfort. Currently, health is considered a multidimensional and multilayered phenomenon among fundamental human rights which is a necessary condition for fulfilling social roles. People can be fully active when they feel healthy and society considers them healthy. In this context, health will be categorized in the ranks of social values, and the biological variables will not be sufficing to provide a comprehensive definition of health. The World Health Organization (WHO) provided the definition of health in Geneva in 1948 that can still be used in health-related theories. This definition encompasses complete physical, mental, spiritual, and social well-being and not merely the absence of disease. For the first time in the world, this definition included social health alongside psychological and physical health. Moreover, the WHO describes differences in health status or the social determinants of health in various strata of society as inequalities. When these inequalities are avoidable, it refers to them as injustices [2].

Although the impact of social determinants of health has been recognized for centuries, the inherent and genuine interaction between social trends and health and disease has recently been raised [3]. The evidence suggests that the utmost burden of diseases and the most pronounced health inequalities in the world arise from social determinants [4]. The social conditions and determinants influencing people’s health are diverse and complex [5]. Social determinants of health can be defined as the conditions in which people are born, grow, live, work, and age, which are influenced by stronger forces such as economic factors, social policies, and political issues [6].

Social determinants of health contain the social factors that promote or weaken individuals’ health and the underlying processes of these factors [7]. The importance of social health is such that individuals who possess it can better cope with problems arising from fulfilling their primary social roles [8]. It must be acknowledged that communicable diseases are becoming more of a social problem than just healthcare issues [9]. Therefore, professionals should particularly consider the differences in social and economic factors and their impact on individuals’ health in emergency health conditions [10]; so that the disadvantaged population does not endure a heavier burden of health emergency as for example pandemics. It should be noted that people from low socioeconomic backgrounds experience worse health conditions and shorter lifespans than those counterparts from higher socioeconomic backgrounds [11].

One of the most significant pandemics in human history is the COVID-19 that has rapidly spread with a high mortality rate. The virus was first discovered in China in late 2019 and has since become one of the most challenging human trials in modern history. Studies have demonstrated the impact of this pandemic on human social factors. As the number of confirmed cases of COVID-19 continue to rise, the virus with negative effects on health systems and increasing the fatal rate has shaken the foundations of the global economy and led to sustainable geopolitical changes [12]. This infectious disease threatens the physical health of societies and, in some cases, leads to fatalities. It also affects both physical and mental determinants on health because it generates uncertainty and confusion among people. Consequently, it imposes unbearable psychological pressure, such as stress, anxiety, depression, and grief, on affected societies [13]. The emergence of a public health crisis like the COVID-19 pandemic can significantly represent roles of social determinants of health on contagious, spreading the infection, and the impact of the pandemic on social health factors. This health crisis even penetrates people’s living conditions and lifestyles to the extent that in-depth changes occur even after the pandemic ends. It is essential to acknowledge that lifestyle is directly related to people’s health [5], therefore, special considerations should be given to the changes of people’s lifestyles after the COVID-19 pandemic ends.

The review of the literature has revealed that most of the studies have considered the medical aspect of this pandemic such as background, prevention, symptoms, risks, complications, treatment, and the psychological and psychiatric effects of the disease [14–16]. Other studies in this field explored disease patterns based on social factors in an infected area [16], effects of bioenvironmental, social, and political approaches on the performance of some centers during the COVID-19 pandemic [17], and understanding of the disease and some social factors such as perceived social support [18]. To the best knowledge of the authors, there is not any study considered the social determinants of health comprehensively. While such study may enlighten the professionals of some hidden factors that could change the medical and health outcomes.

Considering the paucity that we felt in the studies, the research team decided to conduct a qualitative study to speculate the social determinants of health during and after the COVID-19. The main objective was to identify and analyze the various social factors impacting health and to recognize their subgroups. By determining these factors, this study may provide essential information to social and healthcare policymakers. They will be able to use this information in strategic planning to enhance society’s lifestyle and overall health indicators during and after the pandemic of COVID-19. The research team hypothesized that all social determinants on health are interacting with each other and a comprehensive perspective regarding the social determinants of health will provide effective solutions for improving community health.

## Materials and methods

**Diagram 1:**
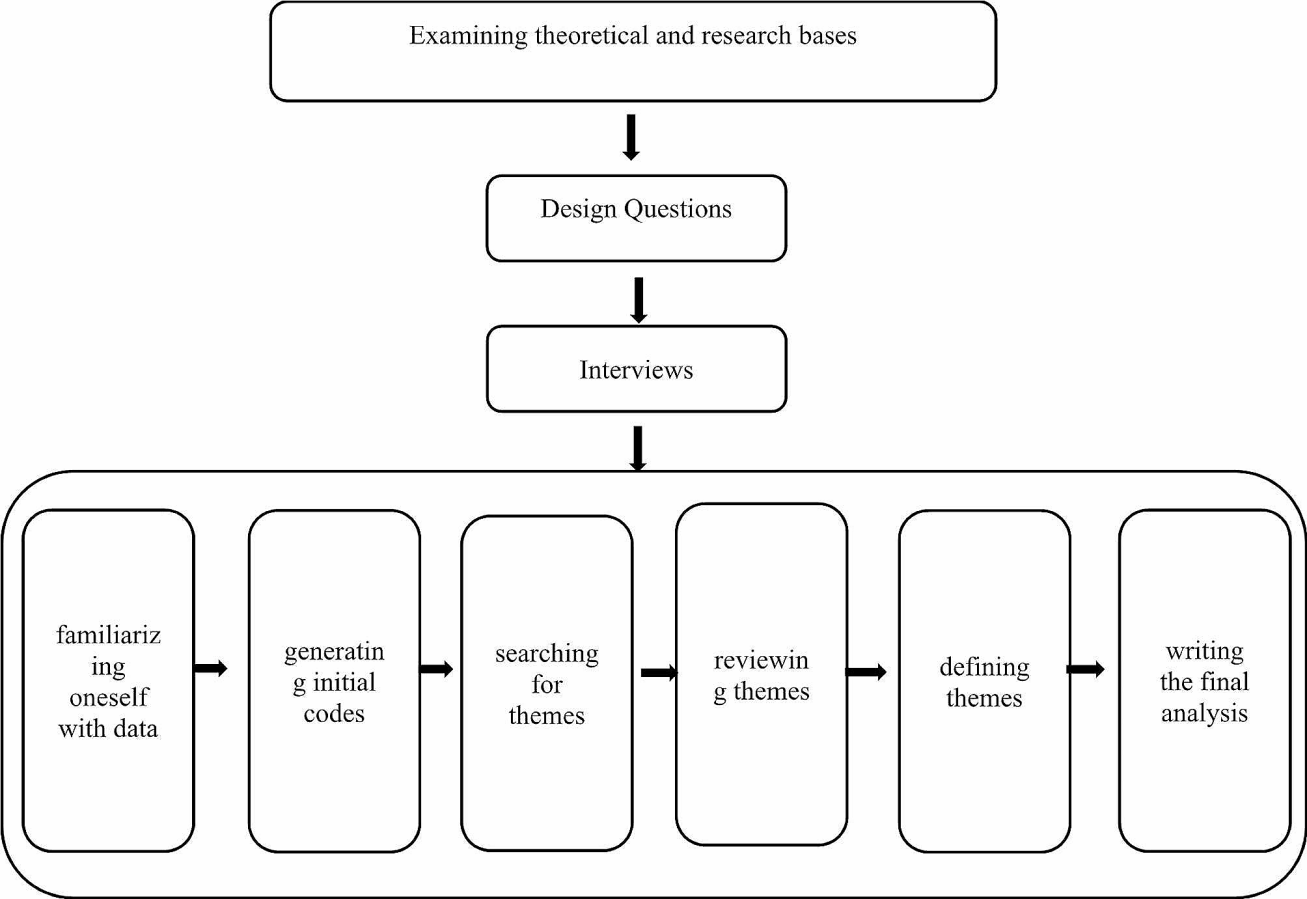
The conceptual framework of the qualitative study of determinants of social factors affecting health during and after Covid-19

The present study had qualitative design. Qualitative research involves studying clients in their natural environments and interpreting phenomena based on the meanings that people attribute to them. In qualitative approach, often conducted through interviews, the aim is to obtain the perspectives and interpretations of the study participants and represent and convey these findings [19]. Regarding to the aim of the present study, the research team chose this method to examine and identify the social determinants of health during and after COVID-19. Therefore, the present study can be considered as a thematic analysis type. Thematic analysis is a method for analyzing qualitative data and is one of the cluster methods that focus on identifying patterns of meaning within a dataset. Unlike many other qualitative methods, thematic analysis is not tied to a specific epistemological or theoretical perspective, making it a flexible approach. The goal of thematic analysis is to identify themes that are patterns in the important and interesting data for the researcher. Braun and Clarke introduced a six-stage framework for conducting thematic analysis, which includes familiarizing oneself with data, generating initial codes, searching for themes, reviewing themes, defining themes, and writing the final analysis [20, 21]. Since we chose to have thematic analysis, the research team followed Brown and Clarke’s six-stage framework. In diagram 1, the steps toward aim of the present study have been displayed.

### Design questions

With the aim of doing the study, the research team started by review of the related literature and upstream documents. In this review, factors, components, and indices of social determinants on health were identified and proper questions to be conducted in interviews were designed. three questions (and exploratory ones), as outlined in Table 1, were used in this study.

**Table 1:** Semi-structured interview guide questions

Number	The questions
1	What do you think about the dimensions or components that the social determinants of health have?
2	How effective do you think the social determinants of health are during COVID-19?
3	How effective do you think the social determinants of health in the post-COVID will be?

### Interviews

To enrich and strengthen the data obtained from the literature, and to develop the conceptual model, expert opinions and specialists’ insights were collected through semi-structured interviews. The sampling method was started by purposeful and non-random (according to Teddlie & Tashakkori, 2023) [21]. To continue the study, the research team undertook the snowball method. Firstly, the research team started interviewing those experts who have been well-known in this field and had all inclusion criteria (to be familiar with Covid-19 and their previous research on the impact of social factors on the occurrence of disease). When the interview was finished, the interviewer asked each interviewee to introduce any other expert that had the mentioned inclusion criteria. Each interview was transcribed immediately by the interviewer. In both groups (scientific and clinical), the interviews were continued until in the last 2–3 interviews no further information was obtained (data/theoretical saturation). The statistical population were two groups. The first group (scientific) was those experts and specialists who had sufficient knowledge in the field of health, i.e., authored books or articles related to prevention, treatment, or health promotion, or those with academic qualifications in this field. The second group (clinical) included individuals who were active in the health field i.e., familiar with health issues, actively engaged in education, research, healthcare, or related fields for the preservation and promotion of health (details of each participant have been presented in Table 2).

**Table 2:** A synoptic table presenting the panel

Number	Gender	Job position	Certificate	Cooperation in related research centers	Expertise			Interview place	period of time (minutes)
					Science	Clinical science	other		
1	Female	non-faculty	PhD	✓	✓			in person	60
2	Female	non-faculty	PhD	-		✓		in person	50
3	male	Academic staff	PhD	-	✓			in person	60
4	Female	Academic staff	PhD	✓	✓			in person	53
5	male	Academic staff	PhD	✓	✓			in person	50
6	male	Academic staff	PhD	-	✓			in person	48
7	male	Academic staff	PhD	✓		✓		WhatsApp	50
8	Female	Academic staff	PhD	✓	✓			WhatsApp	46
9	male	Academic staff	PhD	-	✓			WhatsApp	50
10	male	Academic staff	PhD	✓	✓			WhatsApp	55
11	male	non-faculty	a professional doctor	-		✓		in person	50
12	male	Academic staff	PhD	✓	✓			WhatsApp	52
13	Female	Academic staff	PhD	✓		✓		in person	50
14	male	Academic staff	PhD	-	✓			WhatsApp	56
15	Female	Academic staff	PhD	-		✓		WhatsApp	50
16	Female	non-faculty	a professional doctor	-			✓	WhatsApp	50
17	male	Academic staff	PhD	-	✓			in person	60
18	male	Academic staff	PhD	✓		✓		WhatsApp	58
19	male	Academic staff	PhD	-		✓		WhatsApp	55
20	Female	Academic staff	PhD	-		✓		WhatsApp	50
21	male	Academic staff	PhD	✓	✓			WhatsApp	50

All interviews were administered nationally (including Tehran, Semnan, Khorasan, Orumiyeh, Kashan), between November 2022 and February 2023. To make it possible, the interviewees were able to choose their mode: face-to-face or online (through social messaging apps). Each interview lasted for 45 to 60 min. In face-to-face interviews, the interviewer took notes and recorded whole sessions to be able to do a recheck and have a full transcription. However, for those who chose online mode, the interviewees wrote their answers and sent them back to the interviewer. In both conditions, the interviewers contacted the participants when any response was vague or problematic. Additionally, if a specific point was raised by a participant, in next interview, the interviewer would emphasize on that point to get a clearer response [22].

### Braun and Clarke’s six-stage framework

When each interview was transcribed by the interviewer, the six-stage of thematic analysis was started. Main parts of this process were identifying and encoding semantic units (Basic level), classifying the units into broader categories (Organizing level), and finally, extracting main themes (Comprehensive level). The researchers conducted essential informant confirmations, expert reviews, and document analysis to enhance the data’s reliability. The researcher used manual coding to analyze data. Redundant and excessive codes were removed, and the process continued until broader categories and extensive components related to health were identified.

### Evaluation of study rigor

The research team employed various methods to achieve credibility, dependability, confirmability, transferability, and authenticity. These included member checks, granting participants the opportunity to review and validate or remove interview data, assessing how coding was performed by the research team and individuals familiar with the research methodology (external check), obtaining informed consent, and providing transparent explanations of the research process for all participants, and providing rich data descriptions to ensure a clear understanding of the research process. A sampling method with maximum diversity was also used, and prolonged engagement in the field was maintained. The researcher tried to be accurate in sampling, documenting, data collecting and analyzing, and using the participant-feedback method in addition to quality criterion to make the obtained data valid and reliable.

### Investigator triangulation

To evaluate the credibility of the findings, three people including a pulmonologist, an internal specialist, and a person active in social science studies transcribed the same 20% of all interviews independently. The main researcher asked them to analyze and interpret their transcriptions according to the Braun and Clarke’s approach without prior discussion or collaboration among them. The research team compared all transcriptions, resolved the discrepancies, and reached to a consensus through point-by-point method and reached approximately 90% of agreement among analyzers.

## Results

This study aimed to find social determinants of health during and after Covid-19 through a qualitative approach. Interviews were running with 21 experts with an average age of 44 years (eight were female). Eight had clinical experiences, 17 were academic staffs, and 18 married people. The thematic analysis of the transcriptions induced 10 themes, 33 subcategories, and 132 semantic units.

### Q1: dimensions and components of the social determinants of health

All experts answered this question in full agreement that the conceptual framework of influential factors on health, as expressed by the WHO, is the best model to be used in evaluation of social determinants on health. In their opinions, the social concept mentioned in the WHO’s definition of health could be impressed by at least 10 different themes: (1) Individual factors, (2) Biological factors, (3) Social justice, (4) Social integration, (5) Social acceptance, (6) Social participation, (7) Social adaptation, (8) Social prosperity, (9) Social solidarity, and (10) Spiritual factors.

#### Individual and biological factors

Although in WHO’s definition of health, these factors have their special positions, most of experts in this study believed that age, gender, genetic factors, and mental status impact one’s social determinants on health. Yet, one expert has emphasized the effect of health literacy and media literacy on social determinants on health. They elaborated the mental status factors in different concepts such as the influence of self-efficacy, personality type, interpersonal relationships, and emotional well-being. Besides, the lifestyle of the people could significantly affect their health; even if all conditions for health were met, but then people were not following a healthy lifestyle, their health could still be in danger. Furthermore, most experts believe in the influence of biological factors, such as environmental conditions, on individuals’ health.

#### Social Justice

This themes was extracted from three different policies: economic, government, and societal general rules and regulations. In Table 3, details of the basic units, organizing levels, and comprehensive theme have been presented.

**Table 3:** Explanation of the theme of social justice affecting health

Overarching themes	Themes of organizer 3	Themes of organizer 2	Themes of organizer 1	Basic themes
social justice		Economic policies	Income	Sufficient, insufficient
			Unemployment	periodic, permanent
			employment	skills, job identity, job importance, job independence, job feedback, job security, telecommuting
			Economic situation	Prosperous, non-prosperous
		Government policies	Political participation	Participation in elections, participation in ceremonies
			Civil organizations	People’s institutions, associations, charities
			Communication with organizations	Private organizations, international organizations
	Public policies		Nutrition	Access to proper food, healthy food
			Violence and delinquency	violence at home, violence in the workplace, violence in society, criminal record
			Transportation and traffic	Availability of public transportation, availability of private transportation, use of private car
			Education	primary, secondary, high school
			Access to health services	preventive services, primary health services, medical services, advanced medical services, health insurance
			Organizational status	Organizational support, organizational trust, organizational health
			the environment	Drinking water condition, weather condition, extreme heat and cold, noise
		Housing	place of residence	City, village, suburb, capital
			Instability	Frequent movement within or between cities
			Cohabitation	Alone, with family, with relatives, with friends
			Housing type	Personal, rental, dormitory
			Housing quality	Sufficient space, suitable for the age and physical condition of the residents, acceptable distance to the work environment, adequate ventilation and light, close to health and treatment centers.
		Culture and social values	Religion	The official religion of the country, religious minorities
			education	Primary, middle, secondary, and higher education
			nationality	Persian ethnicity versus other ethnicities (Kurd, Baloch,…)
			Migration	Migration from country to country
			gender Discrimination	Selection of people based on gender
			Language	Persian versus other languages (Turkish, Arabic,…) and dialects (Gilaki, Mazani,…)
			Demanding Culture	Logical and legal demand - illegal demand
			Social norms and attitudes	Religious norms - legal norms

#### Social Integration

The second theme was social integration which was a consolidation of four different organizers (level 2) as displayed in Table 4. Experts (1, 9, and 11) said “the acceptance of the social norms, avoiding of breaking the law, and committed to obey the rules are effective in social health; social health is necessary for the health of the members of the society”. Experts 3 to 7 expressed: “family making is the cause of social health”. Even, expert 11 believed: “being employed is effective in health”.

**Table 4:** Explanation of the theme of social integration among social factors affecting health

Overarching themes	Themes of organizer 2	Themes of organizer 1	Basic themes
social integration	dependency	Accepting the norms of the society	respecting the norms of the society, observing the norms of the society
		Responding to the expectations of others	Respecting the rights of family, colleagues, community members
	Commitment	Refrain from breaking the norm	Awareness of the norms of the society, compliance with the preservation of the norms of the society
		Implementation of conventional activities	Awareness of one’s own and others’ rights, implementation of appropriate activities to respect one’s own and others’ rights
	Busy	employment	Spending less time, more time to work
		family Making	family and spending time with your spouse, spending time with children
	Belief and Faith	Compliance with the rules	Knowledge of society’s laws, compliance with rules in the workplace and society
		Compliance with cultural principles and rules	Knowledge of cultural principles and rules, respect for cultural principles and rules

#### Social Acceptance and Participation

According to the experts, “social judgment, whether in the form of acceptance, rejection, or neutrality, significantly impacts people’s health”. Also, “conformity and social influence of people has an effect on the health of society and consequently on the people’s health”. “The participation of individuals in society, both formally and informally, reflects the health and dynamism of the community”. In Table 5, the thematic analysis regarding these two themes have been displayed.

**Table 5:** Explanation of the theme of acceptance and social participation of social factors affecting health

Overarching themes	Themes of organizer 2	Themes of organizer 1	Basic themes
Social Acceptance	Social influence	Factors affecting social impact	Persuasion, exchange, inspirational attractions, legal methods, pressure, cooperation, informing, favoring, consultation, personal attractions, coalition
		Social perception	Society culture, physical environment
	conformity	weak	Anomy, alienation, social indifference, social alienation
		Strong	Alignment with society’s values, social order, acceptance of collective images
	Social judgment	Reception area	Accompanying the values ​​of society
		field of exclusion	Coping with society’s values
		Neutral domain	Indifference to society’s values
	Individual attitudes	Acceptance of pluralism with others	Accepting the existence of different attitudes of people in society
		Trust in the inherent goodness of others	Believing that people in society are good, not being pessimistic about people
		A positive view of human nature	Valuing human beings, accepting the usefulness of human existence despite having some defects
Social Participation	Formal or institutional partnership	Governmental	Sharing the power of the people, allowing the people to control their own destiny, opening the opportunities for development to the people
		NGOs^*^	Private, semi-private
	Informal partnership	Social perception affected by internal characteristics	A person’s values ​​and attitudes, a person’s personality, a person’s motivation, a person’s experience
		Social perception affected by external characteristics	Structural beauty, intensity, size, context, repetition, movement and change, novelty, familiarity with concepts
		Social perception affected by the state of society	Culture, physical environment
^*^ Non-government organizations			

#### Social Adaptation

To reach this theme, the basic units went through three levels of organizing (Table 6). Ps 2, 5 and 8 said: “Crime, aggression, superstitions, irresponsibility, breaking the law and people stress in society are the marks of lack of health in the society”. P13 said: “Charitable donations, being responsible, and law-abiding are those preventive factors that protect the health in the society”.

**Table 6:** Explanation of the theme of social adaptation of social factors affecting health

Overarching themes	Themes of organizer 3	Themes of organizer 2	Themes of organizer 1	Basic themes
Social Adaptation	Consistency and uniformity	Active compatibility	Positive	conformity, legalism, innovation, social compatibility, universality
			negative	particularism, ritualism, anomie, aggression
		Passive concordance	positive	obedience, adopting silence to take care of our beliefs
			negative	Isolation, depression, despair, secrecy, silence
	Integration and participation	Active	Positive	Cooperation, healthy competition, responsibility
			negative	Ethnicity and sectarianism, rebellion, unhealthy competition, coercion, looting, destruction of others’ character
		Passive	Positive	Coexistence, homogeneity
			negative	Avoidance of responsibility, opportunism, escape
	Acculturation	Active	Positive	Faith and asceticism, legality
			negative	Delinquency, deviance, hypocrisy and deception
		Passive	Positive	Tolerance, imaginative idealism
			negative	Betrayal, fatalism, flattery, anonymity, superstition, distance between speech and action

#### Social Flourishing

A society would flourish if “social happiness and vitality” according to Ps 3, 7, and 14 in addition to “meritocracy, the development of people’s self-esteem, and people’s welfare” Ps 10, 16 and 17 as well as “a sense of community security” based on the experts’ opinions existed. In Table 7, this specific theme along with its subcategories and basic units have been presented.

**Table 7:** Explanation of the Theme of social Flourishing from Social Factors Affecting Health

Overarching themes	Themes of organizer 2	Themes of organizer 1	Basic themes
Social Flourishing	A sense of social security	Respect for individual freedoms	choice of housing and accommodation, correspondence and communication, commuting
		privacy	Physical, informational, internet
		Trust in the government	Honesty of governance against corruption, satisfaction with security and dealing with crime, confidence in governance, response to governance, justice of governance, religious prejudice, financial security, sense of social mobility and satisfaction with educational and health services.
		Meritocracy	Worthy of wanting, worthy of recognition, meritoriousness and meritorious upbringing
		Social Welfare	welfare, financial assistance to disadvantaged people, improvement of people’s living standards, growth of people’s self-esteem, increase of people’s freedom in choosing
	social happiness	Life satisfaction	Sensual and transitory joys, genuine and lasting joys
		Self-esteem	Self-worth and respect, the ability to accept criticism, being oneself, communicating without fear and apprehension, accepting responsibility for one’s life, facing problems, the ability to withstand failure without giving up
		religiosity	Religious beliefs, religious feelings, religious rituals
		Community Relations	Family relationships, relationships with friends, relationships with colleagues
	social Identity	residence	Village, city, megacity, capital
		Ethnicity	Fars, Azeri, Baluch, Kurdish, Lor, Arab
		Religion	Shia, Sunni, other religions
		Job	Employed by the government or the private companies
		Culture	General, subculture (minority)
	Occupational and professional security	Job Satisfaction	Work environment, giving responsibility, fair policies and practices, personal interests and hobbies, caring organization, creativity and leadership, appreciation, gaining respect from colleagues, age, salary, sense of belonging, flexibility
		Fair distribution of income	Labor economics, tax policies, economic policies, policies of labor unions and syndicates, monetary and financial policies, individual abilities of workers

#### Social Cohesion

P_9_ said: “the unity among people and the attraction between the society members are the prerequisites of society health”. In Table 8, this specific theme along with its subcategories and basic units have been presented.

**Table 8:** Explanation of the theme of social Cohesion among social factors affecting health

Overarching themes	Themes of organizer 2	Themes of organizer 1	Basic themes
social Cohesion	Unity with the group	doable	Consultation, understanding
		undoable	Ambition, unwarranted expectations, greed, envy and hatred
	Unity with society	General	Unity and cooperation in community affairs, people’s participation in social organizations, people’s belief, the authority of the government
		Individual	Absence of misplaced pessimism, knowledge and awareness, enjoining good and forbidding evil, voluntary and voluntary participation, honest and sincere service, religion
	Tension between members of society		

#### Spiritual Factors

Considering the WHO’s definition of health, the spiritual aspect should also be considered as all experts said. P_s_ 3, 10, 14, 19 and 20 believed that “spiritual and religious components have an important role in people’s health”. P_20_ believed: “religious and spiritual aspects are subcategories of society’s culture and play roles in people’s health”.

#### Q2

The effective role of social determinants of health during COVID-19.

Most of the experts considered the factors affecting the health of the society during the outbreak of the Corona virus to be similar to the factors affecting health before the pandemic, however, they deemed social factors much more critical during the pandemic. They expressed the influence of appropriate social behavior, without stress and anxiety, a healthy diet, using helpful social media, and physical exercise in controlling the spread of COVID-19 disease (P 1, 2, and 3). P6, while emphasizing the prominent role of social factors in mitigating the pandemic and reducing its casualties, pointed out health education through media, legally mandated physical distancing, and mask-wearing as three main indices contributing to the success of societies in controlling the COVID-19 pandemic, which are all social determinants on health.

Also, some experts considered the pandemic of Corona virus effective on social factors and mentioned that a two-way relationship has been established during the pandemic of Corona virus between the disease and social determinants on health. This relationship could have positive effects including leading to increased empathy, compassion, and cooperative approaches among people; a surge in spiritual tendencies; an increase in respecting each other’s rights; evolution in attitudes of art communities and social science theorists; improvement in media literacy; enhancement of health literacy; an increase in preventive acts, and justifiable therapeutic support. On the other hand, it could have adverse effects, causing public fear; increased hopelessness, family and societal conflicts, and unemployment rate; reduced income, leisure activities, physical exercise, and social interactions; and dissemination of falsehoods. These positive and negative societal effects may result in changes in social factors, once again affecting people’s health (quoted by P 4,5, 6, 9, 11, 18, and 21).

#### Q3

The effective role of social determinants of health after COVID-19.

All experts referred to the specific impact of social determinants of health after COVID-19. P1 said “those who suffered more damages during the pandemic, such as those who lost their jobs, homes, or loved ones, or a decrease in income, are at risk of deteriorating health”. P3 mentioned some achievements would be perceived after COVID-19: “Health proceedings will continue. The use of new technology will persist, leading to reduced traffic, pollution, and road hazards. A transformation in economy, culture, and communications, which is not similar to the pre-COVID era, and these changes will give rise to new social factors affecting health. Individuals will focus on self-care and care for others.”

P4 emphasized: “According to the conditions and extent of crisis management during the pandemic, various psychological, communication, and educational abnormalities have emerged, which will continue for years. These [abnormalities] should be considered as social factors that can compromise health, and appropriate strategies should be devised to tackle them”. P6 said: “initial documentation of the experiences of the Corona era and turning them into artistic and literary works can make these experiences last, and the cooperation of the Ministry of Health and cultural institutions can create a fundamental basis for the introduction of social determinants that affect health in the society. It [the fundamental basis] causes the optimal management of all capacities to achieve justice in health and the mutation of these components”. According to P11 “if social, cultural, and economic factors are optimal, society can more easily return to the pre-COVID conditions”.

## Discussion

Social well-being refers to the ability to interact with others and the environment to establish satisfying interpersonal relationships. Capabilities such as sincere communication with others and managing conflicts in a proper way and ethically over an appropriate and acceptable period of time are among the indicators of social well-being. Respecting others, taking responsibility for the community, and being prepared to spend personal resources for the society and engage in healthy and balanced interactions with others (in a way that neither we nor others are exploited) are considered subcategories of social health. The present study, based on the expert opinions, identified seven social determinants on health, that interacting with personal factors additionally to spiritual factors. Those seven social determinants social justice, social integration, social acceptance, social participation, social adaptation, social flourishing, and social cohesion. Our findings were partially in line with the model presented by Keyes (1998). Keyes presented a multidimensional model for social health, which included social coherence, social acceptance, social contribution, social adaptation, and social actualization [23]. We found two more dimensions that Keyes’ model did not include: social justice and social cohesion. This difference might be a result of the methodology and the concept that each study searched for. Our study had qualitative design assigned to find social determinants of health during and after Covid-19; while Keyes tried to develop a proper scale to evaluate social well-being. It seems the differences of terms in these two studies are superficial, since the definitions and concepts that they include are similar.

The bio and psycho factors of each person can be affected during and after COVID-19, as the experts highlighted in the present study. This is in concert with findings reported by different studies [24–28]. For example, Sher (2021) reported that many COVID-19 survivors experience persistent physical symptoms such as cough, fatigue, dyspnea and pain after recovering from their initial illness; they also experience persistent psychiatric symptoms such as depression, anxiety, and post-traumatic symptoms as well as neurological impairment including anosmia, ageusia, dizziness, headache and seizure. He hypothesized that these symptoms may increase the possibility of suicide or suicidal ideation [24]. Obviously, in a society that a large number of people were and are struggling with bio-psycho symptoms of COVID-19, the social aspect of their lives would be in danger as well (as Einvik and colleagues showed in 2021) [29].

The present study indicated that during and after Covid-19, another dimension that has effects on social health is spiritual factors. These factors have been considered as an important dimension of health [30]. We found spiritual attitudes, spiritual connections, and spiritual behaviors as the most important subcategories that should be considered during and after COVID-pandemic. What the study revealed was in agreement with findings reported by Ghaderi and colleagues (2018). They tried to provide a definition for the spiritual health. By interviewing 21 experts, they were able to find at least three dimensions for spiritual health (including religious, individualistic, and material world-oriented). Their participants distinguished between spiritual health and spirituality; they believed following the spiritual health factors affects the bio-psycho- and social aspects of health [30]. At the time of pandemic, spirituality went through positive and negative perspectives. For example, Büssing and colleagues investigated a specific topic about spirituality “spiritual dryness” during COVID-19 in Iran. They found the spiritual struggle/dryness between 27 and 35% among their participants. The best and positive predictors were usage of mood-enhancing medications, loneliness/social isolation, and praying and negative predictor was being restricted in daily life concerns [31]. At the same time, there are some studies that showed patients with COVID-19 whose spiritual health was enhanced could effectively adapt to their illness [32].

The present study revealed that social determinants of health during COVID-19 were similar to those ones before the pandemic, however, their effects were intensified. Accordingly, Abrams and Szefler (2020) emphasized that the effect social determinants of health have been underestimated during COVID-19. They expand their commentary by the relying on the results of the studies on how the poverty, physical environment (such as, smoke exposure, homelessness), and race or ethnicity might increase the chance of morbidity and mortality with COVID-19. They stated while COVID-19 has been considered as a great equalizer, its outcomes brought up inequalities in social health [33]. Similarly, Singu and colleagues (2020) in a review article took a step further and highlighted the effect of social determinants of health on the outbreak of COVID-19. In a five-dimension model, they showed five social determinants health factors (health and health care, Economy stability, Education, Neighborhood & Built Environment, and social & community context). They also used documents and studies to show how these five factors put people in a hierarchy and vulnerable to the COVID-19 [9]. Hiscott and colleagues (2020) expanded what Abrams & Szefler or Singu and colleagues wrote and added up many details on how social determinants of health changed during COVID-19 and how effective these determinants were [34]. They discussed how strict social distancing measures and home quarantine have resulted in the bankruptcy and closure of many businesses worldwide [29]. Consequently, some individuals have turned to drugs, tobacco, alcohol, gambling, and online gaming to cope with stress, which, in turn, significantly increases domestic violence and sexual abuse [28]. Lifestyle changes during the COVID-19 pandemic, such as increased solidarity, attention to and adherence to health guidelines, and reduction in social gatherings (e.g., weddings), have been observed in various communities throughout the pandemic.

Regarding the influential role of social factors on health after COVID-19 pandemic, according to experts’ opinions, the changes that occurred during the pandemic may persist even after it ends. Studies from other countries reached to similar perspective that danger in social health inequity would last to the post-COVID-19 unless proper policies and interventions to tackle vulnerability in living, education, employment, and poverty design and implement [35]. Lukkahatai and colleagues (2023) took a conserving position about the persistence symptoms of COVID-19 and wrote that “the persistent symptoms of long COVID-19 are less clear”. To be able to provide a proper explanation about the effects of post-COVID-19 on social determinants of health, they used WHO’s model. In this model, to reach appropriate health outcomes two groups of determinants including structural and intermediary are interacting. Details on these two groups are in fact those seven themes that we found in our first step to define the social determinants of health during the COVID-19. The WHO defines socioeconomic and political context and socioeconomic position as the structural determinants and material circumstances, behaviors and biological factors, and psychosocial factors as intermediary determinants. The inter and inter relationship among all these factors and determinants will provide time to resolution of symptoms, health care utilization, quality of life (these three variables as a collection considered to be the ‘health outcomes’) [36]. During the post-COVID-19 period, the number of television viewers and the percentage of activity on social networks are expected to increase significantly [37]. Economic recession, a decline in marriage rates, increased anxiety, and a lack of confidence in the future will reduce fertility rates and lead to consequences such as family disintegration and increased financial pressure [30]. Both during COVID-19 and afterward, significant changes will be observed in social systems and norms.

## Conclusion

Given the wide range of social factors affecting health and their mutual influence on biological and spiritual aspects, we assume each community must identify these factors and plan for their control to overcome social health challenges during and after crisis such as COVID-19. However, this complexity should not stop planners from acting and implementing comprehensive programs, as the effects of pandemics can lead to lifestyle changes during and after, significantly impacting social structures.

### Research limitations

This study faced limitations, such as the lack of transparency in the division boundaries of the dimensions of social factors affecting health and the creation of interferences within each size. The impact of the coronavirus disease on increasing or decreasing the valuation of influential social factors on health (for example, the increased importance of social justice and decreased social participation due to medical quarantine) and access to scattered and fragmented results in global and national studies were also challenges. However, the researchers tried to identify the most influential health factors, particularly social ones, that are important for the Iranian community.

### Application of the Research

The researchers hope that presenting the socially influential factors on health in the post-COVID-19 era can assist health policymakers in making more practical decisions.

### Recommendation

Identifying and formulating an operational plan to reduce the adverse effects of the above interactions is recommended by specifying the areas relevant to long-term, medium-term, and short-term practical programs affected by social factors during the COVID-19 pandemic. Additionally, it will be interesting to find out what social determinants on health were reinforced and what was minored after the COVID-19 pandemic.

## Data Availability

The datasets used and/or analyzed during the current study available from the first author on reasonable request.
