# Correction: Social determinants of health during and after coronavirus: a qualitative study

**DOI:** 10.1186/s12889-024-20653-z

**Published:** 2025-03-13

**Authors:** Farideh Izadisabet, Aliakbar Aminbeidokhti, Sakineh Jafari

**Affiliations:** 1 Department of Midwifery, Faculty of Nursing and Midwifery, Doctoral student of educational management of Semnan University, Semnan University of Medical Sciences, Semnan, Iran; 2 Department of Education Management, Faculty of Psychology and Educational Sciences, Semnan University, Semnan, Iran; 3 Department of Education Management, Faculty of Psychology and Educational Sciences, Semnan University, Central Administration of Semnan University, Campus 1, Semnan, 35131-19111 Iran

**Correction: BMC Public Health (2024) 24:283**

10.1186/s12889-024-17785-7

The original publication of this article contained an incorrect author name. The incorrect and correct information is shown in this correction article. The original article has been updated.

Incorrect

Farideh Izadi sabet

Correct

Farideh Izadisabet

